# The Arctic vegetation is more sensitive to heatwave-induced photosynthetic decline than other climate zones in Europe (2009–2017)

**DOI:** 10.1038/s41598-026-41640-x

**Published:** 2026-03-04

**Authors:** Young-Seok Hwang, Stephan Schlüter, Hyejin Park, Jung-Sup Um

**Affiliations:** 1 Multi-INT & Fusion Division, HANCOM InSpace, Daejeon, 34126 Korea; 2 Department of Mathematics, Natural and Economic Sciences, Ulm University of Applied Sciences, 89075 Ulm, Germany; 3 A3 Architectural Laboratory, Kyungpook National University, Daegu, 41566 Korea; 4 Department of Geography, Kyungpook National University, Daegu, 41566 Korea

**Keywords:** Arctic vegetation, heatwave, climate zones, photosynthesis, Europe, Environmental impact, Natural hazards, Climate-change impacts

## Abstract

This study shows that the photosynthesis of Arctic terrestrial plants is more vulnerable and sensitive to heatwaves’ impacts than plants in other European climate zones. The results of this study indicate distinct effects of heatwaves among different climate zones in Europe (2009–2017). The Arctic climate zone shows a steeper decline in photosynthesis during heatwaves, as revealed by geographically and temporally weighted regression analysis based on biophysical factors such as the Normalized Difference Vegetation Index and Leaf Area Index, compared to arid, temperate, and cold climate zones. As Arctic temperatures continue to rise, it would lead to photosynthetic inhibition from Arctic terrestrial plants, irreversibly turning them into one of the largest CO_2_ sources. The results of this study are expected to be a valuable reference as they provide European-wide quantitative evidence that global warming has caused comparably stronger negative consequences on the photosynthetic physiology of plants living in the Arctic compared to other climatic zones.

## Introduction

Over the past decade, the frequency and intensity of heatwaves (prolonged periods of unusually high temperatures) have increased significantly. This trend is projected to continue throughout the 21st century, with heatwaves becoming more frequent and severe. Across Europe, these heatwaves have been marked by record-breaking temperatures, increasing in intensity and geographic extent. As a result, the duration of summer heatwaves across Europe has doubled, while the number of days with extreme heat has tripled^1^. The heatwave has completely transformed the land cover of the Arctic tundra region, which used to be called permafrost. The density of plants in the Arctic tundra region is rapidly increasing as the vegetation community expands into the former moss area. Previous studies show vegetation cover in the Arctic tundra has increased by more than 40% since 1980^2^. The Arctic terrestrial plants, consisting mainly of tundra and boreal vegetation, account for about 25% of the global vegetated land area. These plants hold approximately one-third of the total carbon in the Earth’s terrestrial plants^3^. Therefore, exploring heatwave-induced suppression of Arctic photosynthetic activities is significant to anticipate the carbon cycle and the stability of high-latitude carbon sinks under committed warming.

Heatwaves have a different impact on the carbon absorption capacity of different European climate zones^4^. To mitigate these impacts, it is essential to understand these variations and develop strategies tailored to each specific climate zone. Previous studies have confirmed that the simultaneous occurrence of heat waves and droughts increases plant stress, significantly decreasing carbon absorption capacity from Antarctica to the European Alps and Bhutan^5,6^. However, these trends are not uniform even across Arctic regions owing to heterogeneity of interaction between vegetation, snow cover, permafrost and climatic factors^7^. For example, the Polar Vegetation Photosynthesis and Respiration Model (PolarVPRM) highlighted that two neighboring Arctic sites (Barrow: dominated by wet sedge, mosses, and lichens; Atqasuk: wetter and slightly warmer) in Alaska showed distinctly different photosynthetic activity due to differences in soil and vegetation types^8^. Euskirchen et al. (2022) emphasized that photosynthetic processes in the Arctic are strongly tied to local variables such as leaf area index, temperature regulation, and light conditions. Arctic vegetation community response can vary substantially across environmental gradients or geographic location^9^.

Tundra plants are adapted to short, cool growing seasons and waterlogged soils under permafrost. When temperatures rise rapidly due to heat waves, plants that grow in their native environment face heat stress. Excessively high temperatures impair the physiological functions of many plants, reducing photosynthetic efficiency. Arctic plants have limited time and resources to recover from heat damage due to their slow growth and short growing seasons. Heatwaves can cause leaf death and tissue damage, decreasing photosynthetic capacity. This, in turn, can severely impact the growth and reproduction of individual plants, ultimately threatening the survival of the entire plant community. Previous studies on the impact of heatwaves on photosynthesis in vegetation across Europe’s polar climates have explored a broad range of effects, including physiological changes such as tissue damage, leaf mortality, and water stress, as well as adaptation mechanisms and ecosystem-level consequences^10^.

Because the process of photosynthetic change differs in durability, complexity, and accumulation across climate zones, it is essential to understand the specific vulnerability of vegetation in the European Arctic to heatwaves compared to vegetation in other climate regions^11^. However, it is difficult to identify specific previous studies that suggest that vegetation in the Arctic region of Europe is more vulnerable to heat waves than vegetation in other climate zones. In particular, no objective evidence has been identified on how the photosynthesis of Arctic vegetation is affected by the heatwave compared to other climatic zones in Europe. Significant knowledge gaps remain regarding the availability of objective evidence to determine the extent to which vegetation in the European Arctic is more vulnerable to heatwaves compared to vegetation in other climate zones.

The carbon exchange between the land and atmosphere is most active during the Arctic tundra’s growing season, as this is the only time photosynthesis and carbon uptake occur^12^. Therefore, identifying the conditions that enhance or hinder carbon uptake during Arctic summers is crucial for understanding the overall carbon balance. Previous studies on Arctic vegetation were mainly cross-sectional studies tracking photosynthetic phenomena at a certain point in time. According to Bhatt, et al.^13^, the heatwave-induced sea ice decline led to a 10–15% reduction in circumpolar Arctic tundra vegetation during the summer. van der Wal and Stien^14^ found a strong correlation (*r* = 0.92) between Arctic plant productivity and summer temperatures across different scales, highlighting that summer conditions are the predominant factor influencing Arctic plant productivity. Previous studies have observed that extremely high temperatures lead to reduced soil moisture and unfavorable growing conditions, which harm the survival rate and distribution of plant species^15^.

Previous studies based on cross-sectional analysis do not reveal differences and changes in photosynthesis by climate zone over time, as they only provide fragmented information at a specific point in time. It is challenging to draw realistic conclusions about how the recent European heatwave affects the photosynthetic activity of Arctic vegetation without relying on long-term accumulated data^16^. Furthermore, logistical challenges and limited data availability have historically hampered comparative research across climate regions^17^. Field observations are often localized and may not accurately represent entire climate zones, making it difficult to generalize findings across highly heterogeneous climatic regions​^18^. This study is designed to address these gaps and improve our understanding of Arctic vegetation sensitivity in the context of extreme climate events. By leveraging Geographical and Temporal Weighted Regression (GTWR), we explored spatiotemporal properties of heatwave-induced photosynthetic decline in Arctic vegetation. GTWR uses a spatiotemporal kernel function with mixed spatial and time lag bandwidth. In this type of mixed kernel function, the weight given to a specific data point is calculated based on the geographic distance between the regression point and each data point, as well as the time lag between them. Therefore, this study aims to evaluate the spatiotemporal sensitivity of heatwave-induced photosynthetic decline in different climate zones across Europe, utilizing GTWR. Our study provides empirical evidence to inform the spatiotemporal weighting of key processes and factors involved in heatwave-induced photosynthesis declines in Arctic vegetation. This can help identify heatwaves’ realistic and quantifiable impacts and their primary drivers on localized photosynthetic responses within Arctic ecosystems. Such insights are crucial for improving future atmospheric CO₂ trajectories and associated climate feedback predictions.

## Materials and methods

### Study area

Europe, the second-smallest continent after Australia, includes 18 distinct climate zones, ranging from arid to Arctic. Forests in Europe cover 35% of the continent’s total land area^19^. It is home to a diverse range of plant species, including boreal tundra woodlands, boreal coniferous forests, temperate steppes, temperate continental forests, subtropical dry forests, and more^20^. Given Europe’s diverse climate zones and plant species, terrestrial plants are expected to exhibit varying responses and sensitivities to repeated heatwaves. Repeated heatwaves in European climate zones exert different stress levels on photosynthesis in various terrestrial plants. This affects factors such as exposure temperatures, duration of exposure, tolerance or acclimation capabilities, timing within the year, and soil moisture availability. Consequently, the heat stress experienced by plants in these 18 climate zones provides an opportunity to examine how different response patterns quantitatively influence photosynthetic inhibition, resulting in reductions in carbon assimilation and growth. Thus, the European continent offers an ideal setting for studying the effects of repeated heatwave-induced photosynthetic inhibition in terrestrial plants.

### Köppen-Geiger climate classification

The challenges of spatiotemporal complexity can be addressed by classifying the intrinsic properties of vegetation traits alongside climatic patterns. The Köppen climate classification was initially developed to identify the climates associated with specific biomes by integrating factors such as air mass types, positions within synoptic weather systems, plant hardiness, and evapotranspiration^21,22^. In this study, we utilized the enhanced Köppen-Geiger climate classification map for the present period (1980–2016) provided by Beck et al. (2018)^21^. This Köppen-Geiger climate classification map has a spatial resolution of 0.0083° (approximately 1 km) and is constructed using combined climatic data on air temperature and precipitation from diverse sources (WorldClim V1 and V2, CHELSA V1.2, and CHPclim V1). Climate zones are categorized into five main climate types: Tropical, Arid, Temperate, Continental (Cold), and Arctic. Since the climate zone map provided by Beck et al. (2018)^21^ has a higher resolution (approximately 1 km at the equator) than GOSAT level 4 XCO_2_, there is a mixture of climate zones in specific grids (2.5° × 2.5°) of GOSAT Level 4 XCO_2_. The mixture of climate zones can obscure the actual influences of factors on GOSAT Level 4 XCO_2_ due to interactions between the different climate zones. Therefore, in this study, we present the results of GTWR only in areas with non-mixed climate zones.

### Model description

This study examines the impacts of heatwaves on photosynthetic activity across European climate zones using a dual modeling framework (Geographically and Temporally Weighted Regression (GTWR) and Sine-trend interdependency analysis) (Table 1). GTWR quantifies spatial and temporal variations in the relationship between XCO₂ flux and photosynthetic indicators (NDVI, FPAR, LAI, and Evapotranspiration), revealing regional heterogeneity in carbon dynamics. This study treats heatwaves as broader climatological trends rather than discrete short-term events^23^. Seasonal variability in heatwaves modulates photosynthesis through a couple of mechanisms. Biochemically, carboxylation and electron transport rise to an optimum and then decline as leaves overheat with excessive heatwaves.

Additionally, excessive heatwaves elevate vapor-pressure deficit (VPD), tightening stomatal control and suppressing carbon assimilation even before biochemical limits are reached^24^. Relations between heatwaves and photosynthetic indicators are strongly co-seasonal. Since the GTWR model (XCO₂ to photosynthetic indicators) embeds seasonal heatwave variability, it can conflate cyclic seasonality with heatwave-driven anomalies. While GTWR primarily captures long-term or inter-annual variations in the photosynthetic response to heatwaves, the Sine-trend Interdependency targets short-term, intra-seasonal dynamics—specifically, the temporal persistence of vegetation anomalies during the recovery phase following extreme heat events. The Sine-trend Interdependency approach was used to identify deviations from the normal seasonal rhythm of photosynthetic activity. By removing the sine-shaped seasonal component from NDVI and related indicators, the analysis isolated non-seasonal anomalies attributable to heatwaves. Residual deviations persisting after heatwave events were defined as legacy effects, reflecting the temporal persistence of disrupted vegetation recovery beyond the natural seasonal cycle.

**Table 1:** Comparison of GTWR and Sine-trend interdependency.

Aspect	GTWR	Sine-trend interdependency
Temporal scale	Captures long-term or inter-annual variations in the photosynthetic response to heatwaves.	Captures short-term, intra-seasonal dynamics in the photosynthetic response to heatwaves.
Main Variables	XCO₂ flux vs. NDVI, FPAR, LAI, ET.	LST vs. NDVI, FPAR, LAI, ET (before/after detrending).
Output	Local coefficients and spatial sensitivity maps.	Correlation strength reflecting intra-seasonal patterns of heatwave effects
Strength	Reveals where and how strongly vegetation sensitivity differs among climate zones.	Supports the results of GTWR by clarifying intra-seasonal patterns of heatwave effects

#### Elements of the GTWR model

**Fig. 1:**
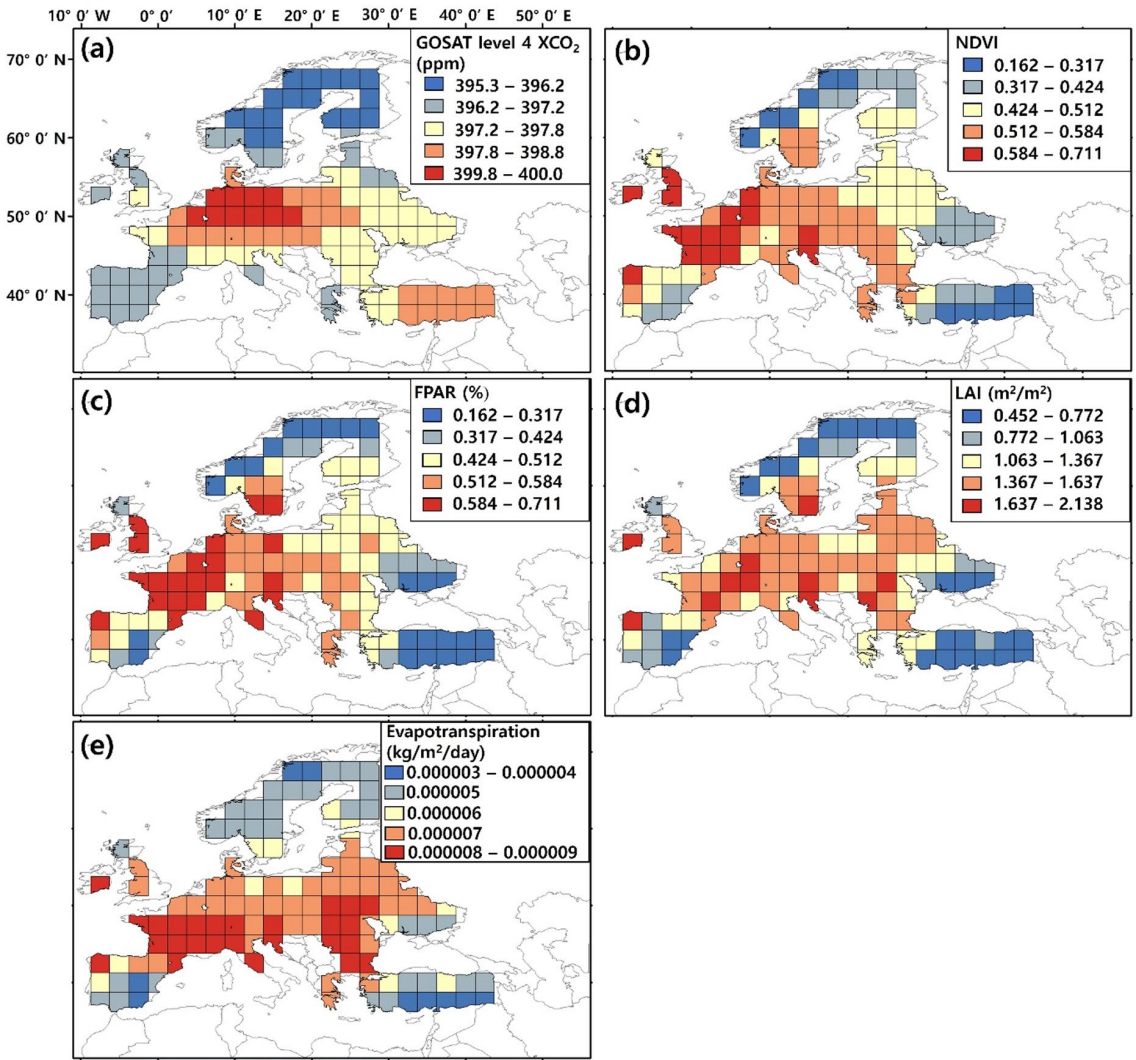
Distribution map of mean monthly data of variables from June 2009 to October 2017. (**a**) GOSAT Level 4 XCO_2_. (**b**) MOD13A2 NDVI. (**c**) MOD15A2 FPAR. (**d**) MOD15A2 LAI. (**e**) MOD16A2 Net Evapotranspiration. The map was generated using ArcGIS (https://www.arcgis.com/index.html) and Adobe Photoshop CS3 (https://adobe-photoshop-cs3-update.en.softonic.com/).

Since the GTWR is a space-time model, it has high explanatory power concerning both spatial and temporal information at a local scale^25^. In this study, we constructed the GTWR model by incorporating normalized variables using the formula $\:(x-\mathrm{m}\mathrm{i}\mathrm{n}(x\left)\right)/(\mathrm{max}\left(x\right)-\mathrm{min}\left(x\right))$, including GOSAT Level 4 XCO_2_ as the independent variable and NDVI (MOD13A2), LAI and FPAR (MOD15A2), and net evapotranspiration (MOD16A2) as the explanatory variables from June 2009 to October 2017 observed from the Moderate Resolution Imaging Spectroradiometer (MODIS) onboard the Terra satellite (Table 2; Fig. 1). The GOSAT Level 4 (L4) dataset comprises two components: Level 4 A (L4A), which provides data on surface CO_2_ flux, and Level 4B (L4B), which offers 3D CO_2_ concentrations derived from L4A. Within the L4B data, CO_2_ concentrations are computed across 17 vertical tiers, ranging from ground level to the upper atmosphere (666 km), with the nearest tier to the ground at 975 hPa^26,27^. Since CO_2_ values near the ground provide valuable insights into changes in sources and sinks, this study used the L4B CO_2_ concentrations at 975 hPa to represent near-surface CO_2_. The timeframe for the L4B CO_2_ dataset spans from June 2009 to October 2017. This dataset provides monthly average CO_2_ concentrations, simulated on a 2.5° × 2.5° geographical grid, and is formatted using netCDF^28^. To align the scales of MODIS indicators with GOSAT XCO_2_, the study integrated MODIS observations by averaging them^29^.

**Table 2:** Descriptive statistics for dependent and independent variables used in GTWR.

Category	Resolution		Min	Max	Mean	STDEV
GOSAT level 4 XCO_2_ (ppm)	2.5​​°	Month	363.59	419.63	397.43	9.02
MOD13A2 NDVI	1 km	16 days	$\:-$0.09	0.81	0.48	0.21
MOD15A2 FPAR (%)	0.5 km	8 days	0.00	0.83	0.41	0.20
MOD15A2 LAI (m^2^/m^2^)	0.5 km	8 days	0.01	4.55	1.23	0.88
MOD16A2 Total Evapotranspiration (kg/m²/day)	0.5 km	8 days	0.0000003	0.00002	0.000006	0.000005

The key vegetation traits that regulate water, energy, and carbon exchange during photosynthesis can be derived from remote sensing observations through four biophysical parameters: Leaf Area Index (LAI), Fraction of Photosynthetically Active Radiation (FPAR), Normalized Difference Vegetation Index (NDVI), and Evapotranspiration (ET) (Fig. 1)^30^. A decline in chlorophyll content reduces the plant’s ability to reflect solar radiation, lowering NDVI values in photosynthetically stressed vegetation^18^. NDVI provides a spectral proxy for canopy greenness and pigment concentration, while LAI quantifies structural capacity for light interception and canopy gas exchange^31^. FPAR indicates the proportion of incoming photosynthetically active radiation (400–700 nm) absorbed by vegetation, directly linking energy input to potential photosynthetic rates and alleviating NDVI’s saturation limits^32^. ET reflects vegetation water use and energy balance, closely coupled with stomatal conductance and carbon assimilation, and is sensitive to heat-induced photosynthetic decline^33^. Photosynthetic indicators (NDVI, LAI, FPAR, ET) represent respective vegetation traits such as greenness, absorbed energy, structural capacity, and water–carbon exchange with distinct physical units and phenological properties. Variance inflation factor (VIF) analysis confirms their differentiation rather than collinear contributions. Thus, NDVI, LAI, FPAR, and evapotranspiration are employed to examine photosynthetic activity.

This study conducted the multicollinearity test by calculating the local Variance Inflation Factor (VIF). Local VIF quantifies locally the degree of multicollinearity between an explanatory variable and the other predictors in a locally weighted regression. It is directly related to the variance of the estimated local regression coefficient for variables. The local VIF values of explanatory variables are presented in Table 3. A local VIF exceeding 10 typically indicates problematic multicollinearity. As shown in Table 3, LAI and Evapotranspiration have mean local VIF values below 10. Mean local VIF values of NDVI and FPAR are slightly over 10, at 14.42 and 19.05. However, GTWR’s adaptive framework mitigates local collinearity. Since GTWR performs locally and temporally weighted regression, the impact of collinearity varies across space and time. In regions, the photosynthetic indicators are possibly more decorrelated due to environmental variability or phenological lags; the model can extract complementary information from each variable. As supported in the literature^34^, multicollinearity primarily affects the stability and interpretability of individual regression coefficients, but not the predictive power or overall model fit, such as Mean Squared Error (MSE) or adjusted R². Even if some degree of collinearity exists, multicollinearity does not compromise the validity of the GTWR model in our analytical context. It does not invalidate the model results or the spatial patterns observed.

While VIF (Variance Inflation Factor) is the standard for global models, the Local Condition Number (CN) is the standard for local spatial models. CN is a diagnostic metric used in Geographically Weighted Regression (GWR and GTWR) to assess the stability of regression coefficients at each specific location. In the context of GWR and GTWR, the academic consensus (referenced in Fotheringham et al., and standard software documentation like ArcGIS and GWR4) is^35^:

- **CN** $\:\le\:$ **30**: The local model is stable. Multicollinearity is not affecting the results.
- **CN > 30**: The local model is unstable. The variables are too correlated at this location, and the coefficients (e.g., NDVI vs. LAI) are unreliable.

**Fig. 2:**
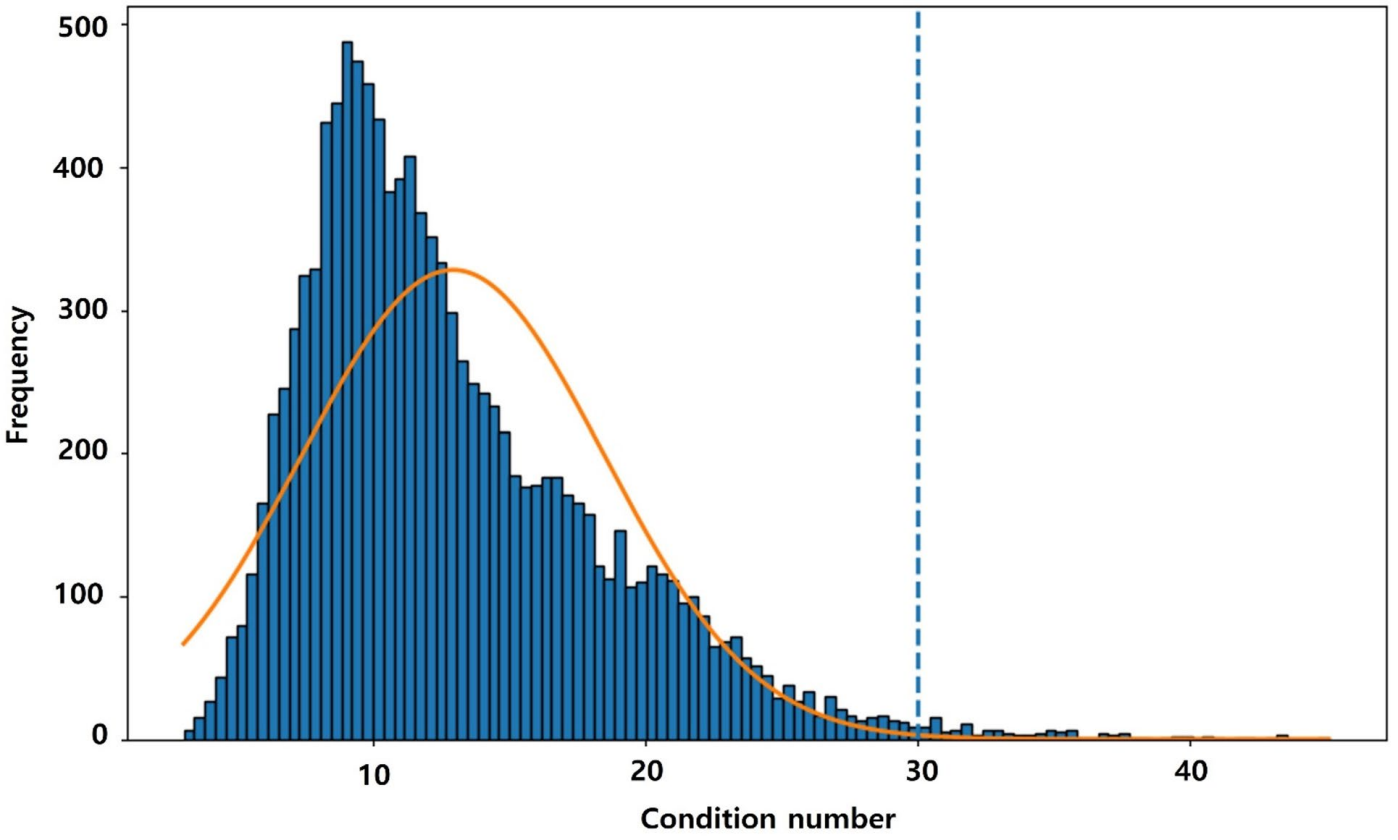
Histogram of local condition number with normal fit. The orange line is the Normal Distribution curve. The dotted blue line is the standard diagnostic criterion (Condition number $\:\le\:$ 30) for local multicollinearity. The figure was created using SPSS (https://www.ibm.com/products/spss-statistics) and Adobe Photoshop CS3 (https://adobe-photoshop-cs3-update.en.softonic.com/).

To confirm the stability of our local parameter estimates, we examined the local condition numbers (CN) for all locations. 98.9% of CN was under 30. Mean CN was also 12.95, below the critical threshold of 30 (Fig. 2). This indicates that local multicollinearity did not significantly degrade the parameter estimation in our GTWR model.

XCO₂ with inversion model is calculated with atmospheric transport and CO₂ emission data (EDGAR, wildfire emission, etc.). The inverted XCO₂ data pertains to emitted CO₂ from mixed sources, leading to coarse information for photosynthetic activities. It needs stronger attribution and should avoid over-interpreting XCO₂ signals that may originate from other sources. While anthropogenic combustion contributes a relatively minor portion of overall variability, XCO₂ anomalies are largely driven by the terrestrial biosphere, as intensified vegetation photosynthesis (gross primary production) during the summer growing season draws down atmospheric CO₂.

Heating-related fossil-fuel use declines in summer in many regions, and the seasonal cycle of power demand and traffic tends to be weaker than the surge in biological uptake. In practice, this yields more frequent negative XCO₂ anomalies over vegetated areas in summer that co-vary with higher photosynthetic uptake^36^, whereas positive anomalies emerge when heat stress or drought suppresses photosynthesis despite the seasonally reduced fossil-fuel emissions^37^. XCO₂ anomalies reflect the combined effects of gross photosynthetic uptake and ecosystem respiration (autotrophic and heterotrophic CO₂ release). Photosynthetic uptake and respiration are sensitive to meteorological variability. During heatwaves and droughts, reduced photosynthetic uptake may coincide with enhanced respiration. Thereby, positive XCO₂ anomalies may also arise from decreased uptake, increased respiratory release, or both. This study focuses on the growing season. Variation of XCO₂ anomalies can be utilized as a milestone for exploring external drivers (heatwaves, droughts, etc.) contributing to photosynthetic activities. However, inversed XCO₂ products are coarser than optical vegetation products. This study aggregates the explanatory variables (LAI, FPAR, NDVI, ET) to the XCO₂ unit scale before GTWR modeling to reduce fine-scale variance induced by coarse information.

Inversed XCO₂ is admittedly coarse, so we model at the XCO₂ native support and harmonize all biospheric covariates to that grid before estimation. In GTWR, inferential capacity is governed not by nominal pixel size but by the local smoother structure. We tune spatial–temporal bandwidths by AIC. Moreover, we quantify model complexity by the effective degrees of freedom (EDF). This measure is based on the actual number of (independent) variables in the model and can be easily computed via the trace of the smoothing matrix^38^. It directly measures the information used by the local fits. Practically, the coarse information content of inversed XCO₂ is handled by resolution-matched design, locally weighted estimation, and bandwidths chosen to balance bias–variance.

By utilizing the monthly deviations in GOSAT XCO_2_ and MODIS observations, this research developed the GTWR model. The GTWR model is defined as follows (Eq. 1):

1 $$\begin{aligned} XCO_{{2i}} = & \beta \:_{0} \left( {u_{i} ,v_{i} ,t_{i} } \right) + \beta \:_{1} \left( {u_{i} ,v_{i} ,t_{i} } \right) \times \:NDVI + \beta \:_{2} \left( {u_{i} ,v_{i} ,t_{i} } \right) \times \:FPAR \\ & + \beta \:_{3} \left( {u_{i} ,v_{i} ,t_{i} } \right) \times \:LAI + \beta \:_{4} \left( {u_{i} ,v_{i} ,t_{i} } \right) \times \:Evapotranspiration + \epsilon_{i} \:, \\ \end{aligned}$$

Where $\:\left({u}_{i}{,\:v}_{i}{,\:t}_{i}\right)$ represent the space–time location of a pixel $\:i$; $\:{\beta\:}_{0}\left({u}_{i}{,v}_{i}{\:,\:t}_{i}\right)$ is the intercept and $\:{\beta\:}_{1}\:to\:{\beta\:}_{4}$ are the local coefficients of the pixel $\:i$ for NDVI, FPAR, LAI, and evapotranspiration, respectively. $\:{XCO}_{2i}$ is the GOSAT level 4 XCO_2_ concentration of the pixel $\:i$. $\:{\epsilon\:}_{i}$ is the regression residual. To calculate the$\:\:\beta\:\left({u}_{i}{,\:v}_{i}{,\:t}_{i}\right)$, we conducted the optimization proposed by Huang, et al.^39^, applying a Gaussian kernel, spatial and temporal distance for estimating the weight matrix $\:W$. The $\:W$ assigns the importance of sample $\:j$ to sample $\:i$ in spatial and temporal scales. The model has high explanatory power (R^2^: 0.793) with a p-value under 0.05 (Table 3). We extracted the local coefficients of the respective biophysical factors from June to August from 2009 to 2017. The changing trends of local coefficient are calculated in monthly growth rate to explore the spatiotemporal sensitivity of photosynthetic indicators on heatwaves over a study period. The growth rates of spatiotemporal local coefficients are computed as a percentage change ($\:\frac{{y}_{t}-{y}_{t-1}}{{y}_{t-1}}\times\:100$).

**Table 3:** Results of GTWR from June 2009 to October 2017.

Climate zone	NDVI	FPAR	LAI	Evapotranspiration	Local *R*^2^
Arid	$\:-$0.05*	$\:-$0.001*	$\:-$0.20*	0.16*	0.80*
Temperate	$\:-$0.03*	0.05*	$\:-$0.25*	0.18*	0.79*
Cold	$\:-$0.10*	$\:-$0.18*	$\:-$0.18*	0.22*	0.79*
Artic	$\:-$0.34*	$\:-$0.22*	0.11*	0.23*	0.78*
Mean Local VIF	14.42	19.05	9.07	6.07	-

R^2^: 0.793* *: p-value < 0.05

#### Sine trend-based interdependence

Photosynthetic activity naturally fluctuates throughout the year, showing seasonal changes (increases and peaks in spring and summer, decreases and minimal activities in autumn and winter). This periodic behavior can lead to misinterpreting normal seasonal variations as heatwave effects. To accurately assess the actual effects of heatwaves on photosynthetic activities, it is essential to analyze and remove underlying seasonality and long-term trends. In this study, a sine-based function is applied to compute seasonality. To account for seasonality, we fit the following sine-based function to all datasets:

2 $$\:\:\:\:\:\:\:\:\:\:\:\:\:\:\:\:\:\:\:\:\:\:\:\:\:Trend\left(Month\right)=A+B*\mathrm{sin}\left(\frac{\left(Month-C\right)2\pi\:}{12}\:\right)\:\:\:\:\:\:\:\:\:\:\:\:\:\:\:\:\:\:\:\:\:\:\:\:\:\:\:\:\:\:\:\:\:\:\:\:\:$$

Note that our estimates are based on a subset of monthly data (specifically, the third quarters (June to August)) from 2009 to 2017. In Eq. 2, parameter ($\:A$) denotes the mean baseline value of the fitted curve. Parameter ($\:A$) is the vertical shift of the sine wave, representing the average level around which the seasonal oscillation occurs. Parameter ($\:B$) is the magnitude of seasonal variation. It defines how strongly the observed values deviate from the parameter ($\:A$) baseline. A larger parameter ($\:B$) produces higher peaks and deeper troughs, while a smaller parameter ($\:B$) flattens the sine wave with less seasonal variation. The Parameter ($\:C$) represents the phase shift, which determines the cycle’s timing by shifting the curve horizontally along the monthly time axis. Units vary across the datasets, so Parameters ($\:A$) and ($\:B$) provide limited insight. However, parameter ($\:C$), representing the seasonal pattern’s phase shift, is informative and highly relevant, especially for interpreting Eq. 2 and the fitted parameters. If, e.g., NDVI and FPAR are perfectly anticyclic, they balance each other out. Both $\:{\beta\:}_{1}$ and $\:{\beta\:}_{2}$ would have low informative value, and their estimates might be skewed after all. Besides, Parameter ($\:C$) is a very rough but aggregated indicator about dependencies on a seasonal level, hence it sheds light on how different photosynthetic indicators, such as NDVI or LAI, for example, evolve in the annual cycle and if this pattern depends on the climate zone.

## Results

**Fig. 3:**
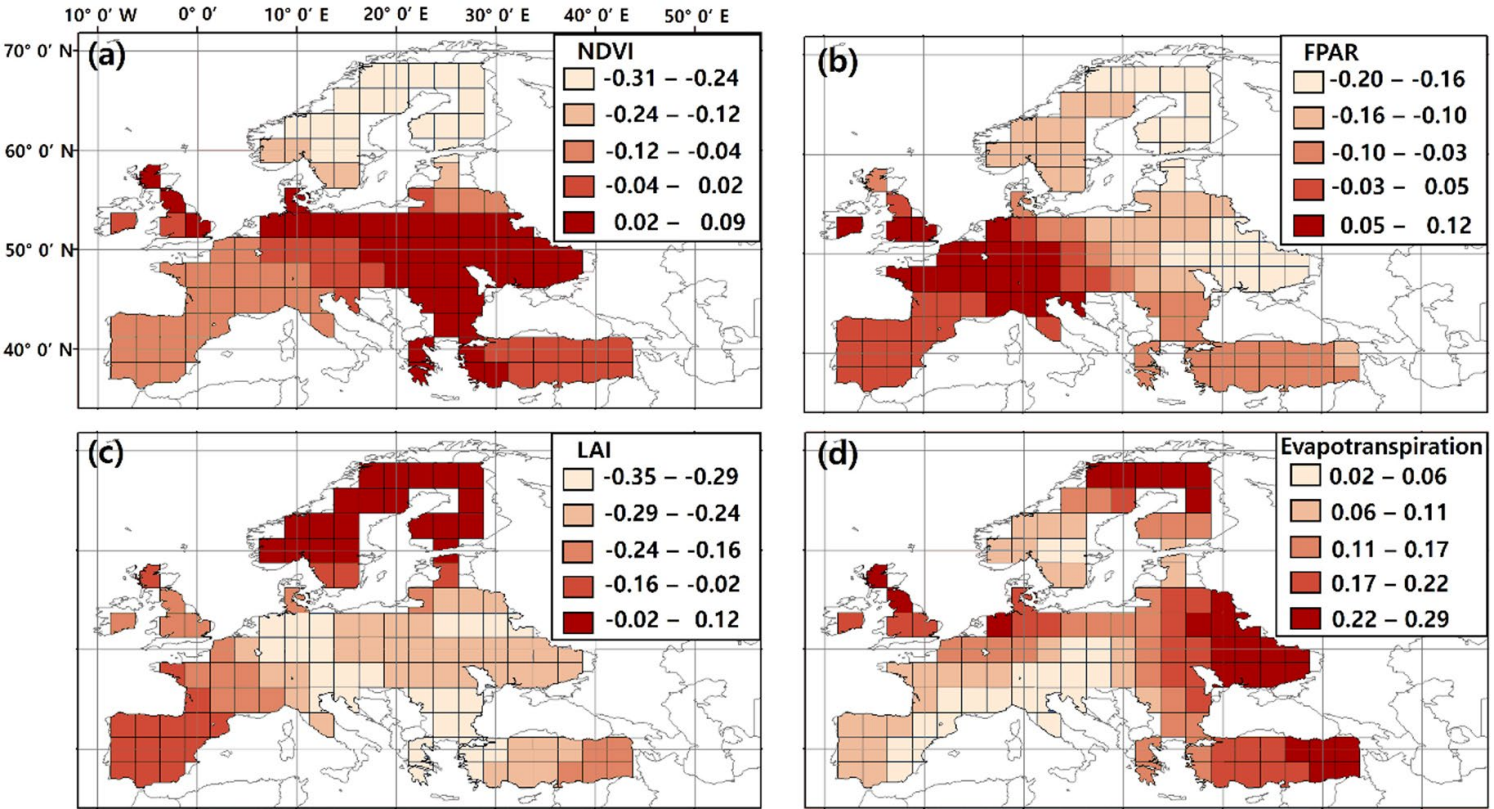
Regional mean GTWR local coefficient of variables in the summer season (June-August) during 2009–2017. (**a**) NDVI, (**b**) FPAR, (**c**) LAI, (**d**) Evapotranspiration. The map was generated using ArcGIS (https://www.arcgis.com/index.html) and Adobe Photoshop CS3 (https://adobe-photoshop-cs3-update.en.softonic.com/).

Spatially varying local coefficients indicate that the influence of biophysical factors on atmospheric XCO₂ differs among climate zones within the European domain during the summers of 2009–2017 (Table 4; Fig. 3). Arid, Temperate, and Cold climate zones show the negative local coefficients of NDVI ($\:-$0.07 to $\:-$0.02), LAI ($\:-$0.20 to $\:-$0.15), and FPAR ($\:-$0.12 to 0.03) except for evapotranspiration (0.11 to 0.16). Decreases of NDVI, FPAR, and LAI and increased evapotranspiration involve the photosynthetic decline in the Arid, Temperate, and Cold climate zones. There are peculiarities between the results of GTWR and sine trend-based interdependencies in FPAR (Temperate and Cold climate zones) and LAI (Cold climate zone). FPAR in the Temperate climate zone has positive and small local coefficients. This indicates that temperate plants manage heat stress well, coinciding with a lower photosynthetic decline. Drought-adapted foliage of Temperate plants copes better with heat stress by maintaining transpirational cooling^40^. The local coefficients of FPAR and LAI are negative in Cold climate zones. This means an increasing photosynthetic activity follows the increase of FPAR and LAI in the Cold climate zone. This is related to the relaxation of cold temperature constraints, increasing broad-leaved tree fraction in European forests and the slow replacement by temperate species^41^.

Contrary, the local coefficients of biophysical factors are heterogeneous and higher than in other climate zones in the Arctic climate zone (Fig. 3; Table 4). Local coefficients of biophysical factors appear positively in FPAR (0.07) and LAI (0.01) from the Arctic climate zone but negatively from other climate zones. In other words, increased FPAR, LAI and evapotranspiration induced by heatwaves drive photosynthetic decline. The response of FPAR and evapotranspiration to heatwaves significantly contributes to the photosynthetic inhibition in the Arctic climate zone. The Arctic exhibits four to fifteen times (NDVI) and 0.5 to two times (evapotranspiration) higher local coefficients than other climate zones. The Arctic climate zone is more sensitive to heatwave-induced photosynthetic inhibition than the Arid, Temperate, and Cold climate zones.

**Table 4:** Descriptive statistics of mean local coefficient of NDVI, FPAR, LAI, and evapotranspiration during 2009$\:-$2017 summer season (June$\:-$August).

Climate zone	NDVI	FPAR	LAI	Evapotranspiration
Arid	$\:-$0.04	$\:-$0.0001	$\:-$0.17	0.11
Temperate	$\:-$0.02	0.03	$\:-$0.20	0.13
Cold	$\:-$0.07	$\:-$0.12	$\:-$0.15	0.16
Artic	$\:-$0.29	0.07	0.01	0.24

**Fig. 4:**
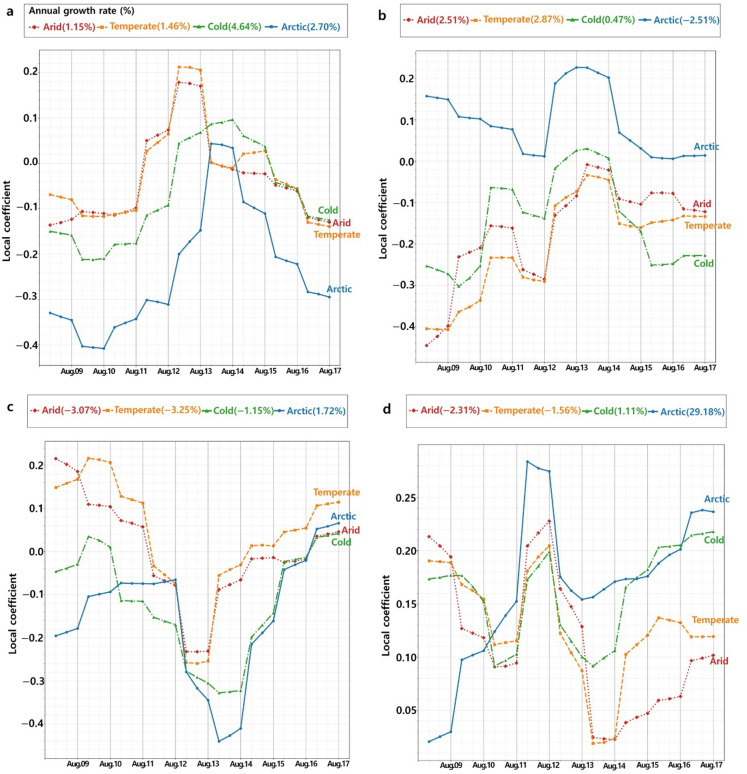
Annual growth rate (unit: %) of the mean local coefficient in Arid, Temperate, Cold, and Arctic climate zones during the summer season (June-August) from 2009 to 2017. a: NDVI, b: LAI, c: FPAR, d: Evapotranspiration. Orange line: Arid climate zone. Green line: Temperate climate zone. Blue line: Cold climate zone. Red line: Arctic climate zone. The figure was created using SPSS (https://www.ibm.com/products/spss-statistics) and Adobe Photoshop CS3 (https://adobe-photoshop-cs3-update.en.softonic.com/).

Figure 4 displays the changing trends and annual growth rates regarding the local coefficient of biophysical factors on photosynthetic activities among climate zones for 2009$\:-$2017. Annual growth rates of local coefficients are shown positively from NDVI (1.15%$\:-$4.64%) and LAI (0.47%$\:-$2.87%) but negatively FPAR ($\:-$3.25% to $\:-$1.15%) from in Arid, Temperate, and Cold climate zones. The annual growth rate of evapotranspiration is shown negatively in Arid and Temperate climate zones ($\:-$2.31% to $\:-$1.56%). The annual growth rate of evapotranspiration shows small changes in the Cold climate zone (1.11%). Positive annual growth rates of local coefficients in NDVI, LAI and evapotranspiration present the constant occurrences of photosynthetic inhibitions in Arid, Temperate, and Cold climate zones. Photosynthetically active radiation (PAR) absorbed by leaves is divided into photochemistry, heat dissipation, and Chl-*a* fluorescence emission. FPAR is less sensitive to heatwave-induced droughts than the NDVI, LAI and evapotranspiration in forested areas^42^. Although FPAR relatively influences photosynthetic activities, the amount of absorbed photosynthetically active radiation for photochemistry (driving electron transport for carbon assimilation) decreases, resulting in an excess of photosynthetic energy^42^. Thus, FPAR shows negative trends in these climate zones owing to decreased forcing weights of NDVI, LAI and evapotranspiration on photosynthetic activities.

The Arctic climate zone exhibits steeper growth rates in local coefficients related to heatwave-induced photosynthetic inhibition compared to Arid, Temperate, and Cold climate zones (Fig. 4). The photosynthetic inhibitions in the Arctic climate zone are an order of magnitude of approximately two to fifteen times more than in other climate zones. In the Arctic climate zone of Europe, most of the local coefficients of biophysical factors are increasing positively as 2.70% (NDVI), 1.72% (FPAR) and 29.18% (evapotranspiration) except for LAI ($\:-$2.51%). The local coefficient of evapotranspiration (29.18%) shows a massive growth rate than other biophysical factors in the Arctic climate zone. Additionally, the growth rates of local coefficients indicate that legacy effects of heatwaves that occurred in previous years act as the accelerator promoting the photosynthetic decline of Arctic plants. The result of this study indicates that responses of biophysical factors to heatwaves contribute largely to photosynthetic inhibition in the Arctic climate zone. In other words, photosynthesis, physiological mechanisms and hardiness of Arctic plants are highly sensitive to the frequency and intensity of heatwaves compared to other climate zones.

The seasonal dynamics of vegetation indices and environmental variables were characterized using a sinusoidal regression model, where Parameter A represents the baseline (mean value), Parameter B determines the amplitude of seasonal oscillation, and Parameter C indicates the phase shift (timing of seasonal peak). These parameters provide quantitative insights into vegetation productivity, seasonal dynamics, and phenological timing across climate zones. Please note that, to stabilize the calibration process, the input data is first standardized by its standard deviation. Hence, Parameters A and B have to be interpreted in this light. For example, if |B| < 1, we have a seasonal pattern weaker than the standard sine swing.

The arid zone exhibits a moderate NDVI baseline (A = 10.56) with subdued amplitude (B = 0.83). Water-limited vegetation response is more controlled by sporadic precipitation than by regular seasonal cycles. The strongly negative phase shift (C = $\:-$3.86) indicates peak greenness in the early growing season before summer drought stress. FPAR and LAI show lower baselines (A = 6.83 and 2.50) with slightly higher amplitudes (B = 1.04). It exhibits that these structural indices better capture seasonal dynamics in water-limited environments. Evapotranspiration shows considerable seasonality (B = 1.20), tracking vegetation phenology. The same can be observed for LST with B = 1.38 (Table 5).

The temperate zone demonstrates the highest vegetation productivity across all zones, with an NDVI baseline (A = 11.28) and a significant seasonal amplitude (B = 1.11) (Table 5). It reflects optimal growing conditions with adequate water and extended growing seasons. The synchronized phase shifts across NDVI, FPAR, and LAI (C = $\:-$1.31 to $\:-$1.43) confirm coherent phenology in temperate ecosystems, with peak greenness in late spring to early summer. Evapotranspiration shows the highest amplitude (B = 1.39) with a delayed peak (C = $\:-$1.69). It is consistent with maximum water demand in mid to late summer when both leaf area and temperature are high.

The cold zone shows substantially reduced baselines (A = 2.62 for NDVI, 1.94 for FPAR, 1.44 for LAI), representing environmental constraints imposed by short growing seasons and low temperatures (Table 5). Yet, the cold zone maintains significant seasonal amplitudes (B = 1.29–1.37) with pronounced seasonal cycles compressed into brief growing periods. The small negative phase shifts (C = $\:-$0.66 to $\:-$0.96) present that vegetation peaks in mid-summer during the warmest months, when the photoperiod is longest.

The arctic zone exhibits the most extreme environmental constraints with the lowest baselines across all zones (A = 1.13 for NDVI, 1.04 for FPAR, 0.92 for LAI) (Table 5). This results from extremely low tundra productivity. While NDVI and FPAR maintain seasonal amplitudes comparable to other zones (B = 1.31 and 1.30), LAI shows markedly reduced amplitude (B = 0.90). This means the limited vertical vegetation structure even at peak season. The phase shifts approaching zero (C = $\:-$0.09 to 0.01) are particularly significant. Arctic vegetation peaks very close to mid-year, when solar radiation is at its maximum. It shows the tight coupling between photoperiod and the extremely compressed growing season.

Three key patterns emerge from the parameter comparison. First, baseline productivity (Parameter A) follows a clear gradient aligning with known patterns of net primary productivity and environmental constraints (Temperate > Arid > Cold > Arctic). Second, seasonal amplitude (Parameter B) remains surprisingly consistent (0.9–1.4) across all zones. Seasonality as a proportion of mean conditions is maintained even in harsh environments, with Arctic LAI as the notable exception. Third, phenological timing (Parameter C) shows a progressive shift from strongly negative (Arid: C = $\:-$3.86) toward zero (Arctic: C ≈ 0). This pattern reveals a fundamental shift in what controls when vegetation peaks. In Arid regions, vegetation must grow early (spring) to avoid summer drought, so water availability dictates timing. In Arctic regions, the extremely short growing season forces vegetation to peak at the time of maximum sunlight (mid-summer).

**Table 5:** Results for Parameter (A, B, $\:\boldsymbol{C}$) of sine-based function.

Climate zone	Parameter	NDVI	FPAR	LAI	Evapotranspiration	LST
Arid	A	10.563	6.827	2.498	3.021	6.996
	B	0.834	1.039	1.042	1.203	1.382
	C	$\:-$3.860	$\:-$2.786	$\:-$2.282	$\:-$3.041	$\:-$0.944
Temperate	A	11.278	6.246	2.709	1.741	8.054
	B	1.108	1.185	1.229	1.385	1.373
	C	$\:-$1.311	$\:-$1.251	$\:-$1.432	$\:-$1.691	$\:-$1.030
Cold	A	2.616	1.936	1.440	1.211	5.460
	B	1.362	1.373	1.292	1.345	1.376
	C	$\:-$0.655	$\:-$0.753	$\:-$0.956	$\:-$1.503	$\:-$1.088
Arctic	A	1.131	1.037	0.916	1.149	4.998
	B	1.309	1.304	0.897	1.295	1.374
	C	$\:-$0.093	$\:-$0.020	0.011	$\:-$1.305	$\:-$0.843

Sine-based trend interdependency frameworks validate regional heterogeneity in photosynthetic sensitivity to heatwave regimes across climate zones. Correlation values are computed based on raw and detrended data (Table 6). The negative correlation values between biophysical factors versus LST at Arid and Temperate climate zones before detrending may result from heatwave-induced droughts. European countries have suffered consecutive heatwave-induced droughts from 2009 to 2017^43^. Plants react to water stress induced by droughts by altering the photosynthetic mechanism of photosynthesis. The stomal closure (decrease of evapotranspiration) is followed by leaf loss (decrease of LAI) and tree mortality (decrease of NDVI). In-situ survey concerning defoliation forests submitted from 27 European countries also reported increasing deterioration in the condition of European forests in 18.9% of the monitoring plots from 2010 to 2018 in response to persistent droughts^19^. Interestingly, the pre-detrended correlation values of Temperate, Cold and Arctic climate zones indicate a distinct negative correlation, opposite to the Arid and Temperate climate zones. Table 6 shows significantly positive correlation values before detrending for the Cold and Arctic climate zones, with the value for Evapotranspiration versus LST being the highest. Positive dependence indicates that repeated heatwaves enhance the biophysical factors in these climate zones. This is likely associated with heatwave-induced water stress. Andreassen et al. (2020) addressed that a surface elevation change and geodetic mass balance were reduced from 817 km^2^ in the 1960s to 734 km^2^ in the 2010s, giving surface elevation and geodetic mass balance reduction of $\:-$15.5 m and $\:-$0.27 ± 0.05 m w.e.a^− 1^ for the 50 years^44^. Arctic climate zone of Europe, the glacier retreat owing to heatwaves negatively impacts drought risk in the growing season due to a runoff decrease of approximately 40%^45^.

By comparing both results, a few interesting aspects are notable. Substantial changes in correlation strength and direction were observed after applying a sine-based detrending approach to remove seasonal cycles (Table 6). Values for Arctic data hardly differ. It indicates only a minor seasonal pattern. The largest influence of seasonality can be seen in other climate zones. Arid climate zone exhibits the weakest and moderately positive correlations between LST and vegetation indicators before (r = $\:-$0.03 to 0.51). After detrending, photosynthetic indicators shifted to negative response (r = $\:-$0.53 to $\:-$0.20). Heatwaves directly suppress photosynthetic activity in water-limited environments, largely due to increased vapor pressure deficit, soil moisture depletion, and stomatal closure under thermal stress in Arid zones. Even after removing natural phenological cycles, the detrimental effects of high temperatures remain evident, suggesting that heatwaves in arid regions primarily exacerbate water stress, leading to significant declines in NDVI, evapotranspiration, FPAR, and LAI. In temperate zones, initial correlations were positive (*r* = 0.78 to 0.89), suggesting an increase in vegetation activity during high-temperature periods. However, after detrending, the correlations shifted to negative values for evapotranspiration, and LAI (*r* = 0.35 to 0.55), while NDVI and FPAR are decreased (*r* = 0.13 to 0.52). Cold regions initially show positive correlations (*r* = 0.87 to 0.97) that decrease after detrending (r = $\:-$0.43 to 0.53). Heatwaves enhance vegetation activity, but the effect is highly conditional in the Cold climate zone. The raw data shows apparent negative relationships in the Cold climate zone. Arctic ecosystems show the strongest and most stable positive correlations with LST (raw: *r* = 0.60 to 0.92; detrended: 0.24 to 0.65) between pre- and post-detrending. It exhibits that LST increases almost universally, leading to a variation of photosynthetic activities in the Arctic climate zone. Small changes from correlation values before and after detrending confirm that this positive response is not seasonal noise but derived from constant effects of LST on Arctic photosynthetic activities. In other words, heatwaves consistently influence Arctic vegetation, leading to higher increases in NDVI, Evapotranspiration, FPAR, and LAI values than other climate zones. The comparison between pre- and post-detrending results of Sine trend-based interdependencies back up the legacy effects of heatwave gradients on the response of biophysical factors among climate zones.

**Table 6:** Results for before and after Sine-based detrended correlation between LST and other biophysical factors in climate zones.

Category		NDVI	Evapotranspiration	FPAR	LAI
Correlation against LST before detrending	Arid	$\:-$0.03	0.33	0.39	0.51
	Temperate	0.78	0.89	0.88	0.79
	Cold	0.95	0.89	0.97	0.87
	Arctic	0.89	0.92	0.88	0.60
Correlation against LST after detrending	Arid	$\:-$0.36	$\:-$0.53	$\:-$0.20	$\:-$0.40
	Temperate	0.13	$\:-$0.29	0.57	$\:-$0.42
	Cold	0.53	$\:-$0.43	0.52	$\:-$0.35
	Arctic	0.60	0.55	0.65	0.24

## Discussion

This study provides empirical evidence that vegetation in the European Arctic is disproportionately sensitive to heatwaves compared with other climate zones. GTWR and Sine-trend analyses consistently indicate persistent legacy effects of heat stress on photosynthetic activity. Arctic vegetation exhibited two- to fifteen-fold stronger responses of NDVI, FPAR, and evapotranspiration to heatwaves, suggesting that ecosystems historically adapted to cold and short growing seasons are experiencing unprecedented physiological stress under rapid warming.

Projections based on GTWR local coefficients (Fig. 4) and cosine-trend interdependencies (Table 6) show that recurring heatwave-induced changes in biophysical factors continuously affect photosynthetic activity, amplifying Arctic vegetation sensitivity. Increased evapotranspiration emerged as a key driver of photosynthetic inhibition. Prolonged heatwaves accelerate permafrost thawing, initially enhancing soil moisture and evapotranspiration but ultimately causing landscape drainage and water deficits^46^. This reduces stomatal conductance, lower leaf water potential, and impaired physiological functioning, decreasing photosynthetic efficiency and productivity. The progressive soil drying and declining evapotranspiration impose chronic stress that alters community structure and weakens the carbon-sink function of Arctic ecosystems. Therefore, the lagged effects of heatwaves on evapotranspiration would be the major drivers of accelerating Arctic vegetation into CO_2_ sources.

While previous studies have reported sustained greening and productivity increases in the Arctic^47^, recent evidence points to a weakening or reversal of this trend, particularly in the European sector^48^ or even reverse, with extensive browning observed particularly in the European Arctic^49^. Rising temperatures and altered precipitation regimes have been identified as key but regionally variable drivers of browning^50^. Constant extreme heat events in the Arctic climate zone possibly accelerate declines in photosynthetic efficiency and ultimately reduce the region’s role as a carbon sink. If Arctic plants transition from carbon sinks to carbon sources due to heatwave-induced evapotranspiration stress, the additional CO₂ emissions could offset mitigation efforts in other regions.

This study also highlights the lagged effects of heatwaves on photosynthetic responses and quantifies spatio-temporal forcing weights for the underlying biophysical processes. Although GTWR effectively captures spatial and temporal variability in vegetation–CO₂ interactions, its local coefficients may partially overestimate photosynthetic inhibition due to concurrent soil carbon losses from permafrost thaw, soil temperature and moisture changes, and climatic factors (precipitation and so on). This is because a substantial amount of carbon is stored in the surface and deeper permafrost layers. Future work should integrate vegetation water-stress dynamics with soil carbon processes to better constrain the feedback mechanisms linking permafrost degradation, evapotranspiration, and Arctic carbon fluxes by incorporating emerging AI-driven geospatial foundation models^51–53^.

## Conclusion

This research highlights the urgent need to address the unclear mechanisms of heatwave-induced photosynthetic inhibition across different climate zones. The results of this study provide objective and tangible evidence of how vulnerable the vegetation of the European Arctic region is to heat waves compared to vegetation in other climate zones. Arctic vegetation experiences a significantly greater decline in photosynthesis compared to other climate zones, primarily due to reduced runoff from melting snow and precipitation combined with increased evapotranspiration. The local regression coefficients for Leaf Area Index (LAI), Fraction of Photosynthetically Active Radiation (FPAR), and evapotranspiration are higher in the Arctic than in other climate zones. In the Arctic, positive local coefficients were observed for LAI (0.01) and FPAR (0.07), whereas other climate zones exhibited negative or weaker correlations (LAI: $\:-$0.20 to $\:-$0.15; FPAR: $\:-$0.12 to 0.03). Additionally, between 2009 and 2017, the Arctic showed the highest growth rates in the local coefficients of key biophysical factors (NDVI, FPAR, and evapotranspiration) compared to all other climate zones. To achieve the Paris Agreement’s goal of net-zero carbon emissions by 2050, greater attention must be given to heatwave-induced photosynthetic inhibition in the Arctic, as it could lead to additional CO₂ emissions that offset reductions from anthropogenic sources.

## Data Availability

The datasets analyzed during the current study are available in the GOSAT Data Archive Service and NASA LAADS DAAC data archive repository maintained at the Japan Aerospace Exploration Agency (JAXA), National Institute for Environmental Studies (NIES), NASA Goddard Earth Science Data and Information Services Center ( https://data2.gosat.nies.go.jp/index_en.html ; https://search.earthdata.nasa.gov/search).
